# Silicone Oil–Silicone Intraocular Lens Interaction

**DOI:** 10.18502/jovr.v21.18149

**Published:** 2026-09-07

**Authors:** Niloofar Piri, Jacob D. Grodsky, Hossein Asghari

**Affiliations:** SSMHealth Medical Group, Department of Ophthalmology, Saint Louis University, School of Medicine, Saint Louis, MO, USA

##  PRESENTATION

**Figure 1:**
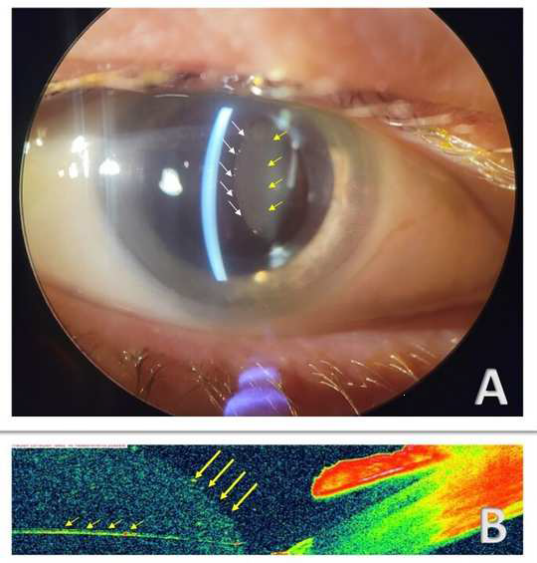
(A) Slit lamp photo demonstrating convex protrusion of silicone oil on the anterior IOL surface. White arrows point to the oil surface, and yellow arrows point to the anterior IOL surface. (B) Anterior segment OCT demonstrating a mildly hyper-reflective homogenous convexity anterior to the IOL surface consistent with silicone oil. Long arrows point to the oil surface, and short arrows point to the hyper-reflective anterior IOL surface.

A pseudophakic 64-year-old patient presented to our retina clinic for follow-up 1 year after undergoing retinal detachment repair with placement of 5000 centistoke silicone oil at an outside institution. Since surgery, he reported a gradual decline in vision.

At the time of presentation, visual acuity was hand motion, intraocular pressure was 22 mm Hg, and anterior segment examination revealed a three-piece intraocular lens (IOL), located in the sulcus, with a convex layer of silicone oil protruding from the anterior IOL surface [Figure 1A]. Retro illumination demonstrated a good red reflex despite several silicone oil droplets adherent to the posterior IOL surface. However, indirect ophthalmoscopy revealed no view to the posterior segment despite the excellent red reflex.

Anterior segment optical coherence tomography (OCT) confirmed these examination findings, showing a large silicone oil bubble protruding anteriorly from the IOL [Figure 1B]. While the patient was unaware of the type of IOL implanted during prior cataract surgery, there was strong suspicion that it was made of silicone and was interacting with the silicone oil from the posterior segment. Surgical records subsequently confirmed the IOL to be a Bausch & Lomb LI61AO lens, which is made of silicone.

The patient underwent surgery to remove the silicone oil as well as exchange of the silicone IOL for a three-piece acrylic IOL in the sulcus. On postoperative day one, visual acuity improved significantly to 20/60.

##  DISCUSSION

The interaction between silicone oil and silicone IOLs has been well described. This process is typically gradual, with silicone oil droplets adhering to the IOL surface and manifesting as retrolental bubbles that scatter light and degrade visual quality.^[1,2]^ Straylight and optical aberrations from this interaction can cause progressive vision decline, even when the posterior segment is otherwise stable.^[2]^

Our case illustrates an extreme manifestation of this phenomenon. Silicone oil migrated to the anterior surface of the lens, where it created a large convex protrusion. We propose that this bubble functioned as a very high plus power lens, producing a profound myopic shift. This likely explains both the patient's severe decline in vision and the inability to visualize the posterior segment with indirect ophthalmoscopy, despite a good red reflex.

Following surgical removal of the silicone oil and IOL exchange with an acrylic lens, visual function improved rapidly. This highlights not only the reversibility of this complication but also the critical importance of IOL material choice in surgical planning.

Given the myriad of IOL choices currently on the market, this case underscores key considerations for both cataract and retina surgeons. Retina surgeons, in particular, should be proactive in obtaining IOL information preoperatively and consider IOL exchange at the time of retinal surgery if silicone oil tamponade is anticipated in a patient with a silicone IOL. Such precautions can help prevent long-term, vision-threatening complications.

##  Financial Support and Sponsorship

None.

##  Conflicts of Interest

None.
